# Computational model for lipid binding regions in phospholipase (Ves a 1) from *Vespa* venom

**DOI:** 10.1038/s41598-023-36742-9

**Published:** 2023-06-30

**Authors:** Nawanwat C. Pattaranggoon, Sakda Daduang, Thanyada Rungrotmongkol, Withan Teajaroen, Varomyalin Tipmanee, Supot Hannongbua

**Affiliations:** 1 Programme in Bioinformatics and Computational Biology, Graduate school, Chulalongkorn University, Bangkok, 10330 Thailand; 2 Division of Pharmacognosy and Toxicology, Faculty of Pharmaceutical Sciences, Khon Kaen University, Khon Kaen, 40002 Thailand; 3 Department of Biochemistry, Faculty of Science, Center of Excellence in Structural and Computational Biology, Chulalongkorn University, Bangkok, 10330 Thailand; 4 Faculty of Associated Medical Sciences, Center for Innovation and Standard for Medical Technology and Physical Therapy, Khon Kaen University, Khon Kaen, 40002 Thailand; 5 Department of Biomedical Sciences and Biomedical Engineering, Faculty of Medicine, Prince of Songkla University, Hat Yai, Songkhla 90110 Thailand; 6 Department of Chemistry, Faculty of Science, Center of Excellence in Computational Chemistry (CECC), Chulalongkorn University, Bangkok, 10330 Thailand

**Keywords:** Biochemistry, Computational biology and bioinformatics, Chemistry

## Abstract

The Thai banded tiger wasp (*Vespa affinis*) is a dangerous vespid species found in Southeast Asia, and its stings often result in fatalities due to the presence of lethal phospholipase A$_{1}$, known as Vespapase or Ves a 1. Developing anti-venoms for Ves a 1 using chemical drugs, such as chemical drug guide, remains a challenging task. In this study, we screened 2056 drugs against the opening conformation of the venom using the ZINC 15 and e-Drug 3D databases. The binding free energy of the top five drug candidates complexed with Ves a 1 was calculated using 300-ns-MD trajectories. Our results revealed that voxilaprevir had a higher binding free energy at the catalytic sites than other drug candidates. Furthermore, the MD simulation results indicated that voxilaprevir formed stable conformations within the catalytic pocket. Consequently, voxilaprevir could act as a potent inhibitor, opening up avenues for the development of more effective anti-venom therapeutics for Ves a 1.

## Introduction

Venom contains small molecules, peptides, or proteins that can cause harm to organisms upon absorption, inhalation, or ingestion, especially when secreted by venomous animals. Venomous arthropods, which are found worldwide, comprise a large proportion of venomous animals^1^. Insects are the most prevalent venomous arthropods, with the Hymenoptera order containing the most insects that cause sting-allergic reactions; however, only three families frequently sting humans: Apidae (bees), Formicidae (ants), and Vespidae (wasps)^2^. Stinging accidents from the Thai banded tiger wasp (*Vespa affinis*) have resulted in fatalities in Southeast Asia^3^. Moreover, rural communities, including those who rely on forests for their livelihoods, such as agriculture, animal husbandry, and foraging, are more likely to encounter dangerous animals, particularly wasps.

The venom of *V. affinis* contains a lethal protein called phospholipase A$_{1}$ (Vespapase or Ves a 1)^3^. Ves a 1 activity not only causes allergic reactions or anaphylaxis, but also disrupts biological membranes^3,4^, causes haemolysis^4^, inflammation^5^, and platelet aggregation, which leads to thrombosis^6^. Furthermore, *V. affinis* species are primarily responsible for the most number of deaths among hymenoptera stings^7,8^. The severity of the stings is determined by the venom to which the patient is exposed. Reports indicate that more than 50 wasps are capable of injecting venom into the patient’s bloodstream, leading to acute toxcity, anaphylaxis, and organ failure. The venom may also destroy the area where the wasps stung the patient, leading to death^7,8^. Epinephrine is the most commonly used medication to treat sting-allergic reactions. However, reducing the risk of anaphylaxis and venom immunotherapy is not recommended for large local reactions caused by Ves a 1^9^.

**Figure 1:**
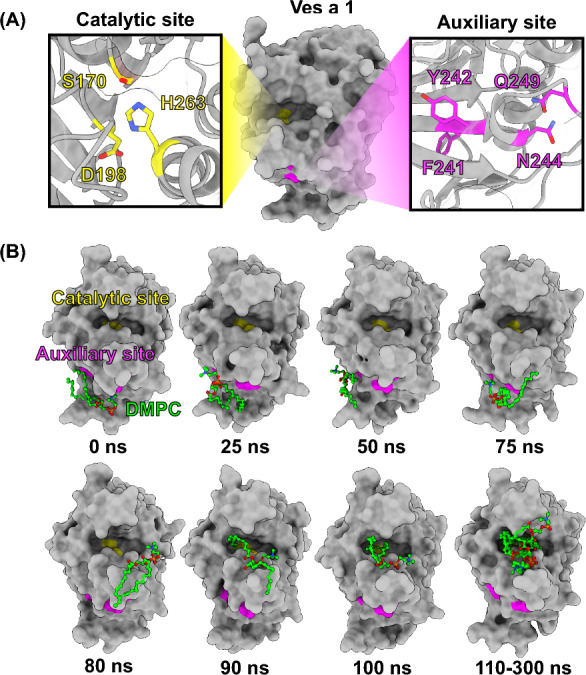
(**A**) The predicted structure of the Ves a 1 protein is shown, with the catalytic triad (S170, D198, and H230) and auxiliary site (F241, Y242, N244, and Q249) depicted as surface representations in green and blue, respectively. (**B**) The transfer of DMPC from the auxiliary site to the catalytic site is illustrated.

Wasps can cause health problems due to their sting, which may lead to severe or fatal systemic anaphylaxis, and direct venom toxicity in severe cases. Among the causes of death, approximately 34% of cases were due to multi-organ failure, with renal failure being the most common complication^10^. Direct venom toxicity causes various signs and symptoms, such as local inflammation and the development of skin haemorrhage or necrotic lesions^11^. Multiple wasp stings can result in hypotension, acute intravascular haemolysis, thrombocytopenia, rhabdomyolysis, acute renal failure, and pulmonary oedema^12^. Currently, there are no consensus guidelines regarding the use of steroids or the initiation of peritoneal dialysis for acute kidney injury caused by insect stings^13,14^. Anti-inflammatory drugs, antihistamines, and steroids are used to treat specific wasp sting symptoms, whereas anaphylaxis cases may require intrathecal epinephrine, intravenous epinephrine, and corticosteroids. However, caution should be exercised as hypertensive patients may be at risk of an acute hypertensive crisis, angina pectoris, or myocardial infarction^12^. Severe symptomatic treatments, such as respiratory support, cardiovascular support, haemodialysis, and haemoperfusion, may also be necessary.

The hymenopteran insect, *V. affinis*, is known for its venom, which contains major protein allergens such as antigen 5, phospholipase A$_{1}$B, hyaluronidase, and protease^5,15^. In 2013, the Ves a 1 protein was discovered in *V. affinis* venom^3^. The protein sequences of Ves a 1 and other related phospholipase A$_{1}$ protein families from different sources share a high degree of similarity with phospholipase A$_{1}$ (vPLA$_{2}$) protein from *V. basalis* and phospholipase A$_{1}$ VesT1.02 (VeT1.02) protein from *V. tropica*, with 93% and 92% similarity, respectively (Fig. S1B). All of these enzymes contain the typical catalytic triad residues Ser-X-Asn-X-His^4,16^ (Fig. S1A,B).

The X-ray crystal structure of vPLA$_{2}$ shows that the $\alpha$/$\beta$ hydrolase fold at the N-terminus is necessary for catalytic activity. The tertiary structure dominants, $\beta$5 and $\beta$9, as well as the lid domain, are important in catalysis. The $\beta$9 and lid domains play a role in substrate recognition and selectivity^4,17,18^. The catalytic triad residues of vPLA$_{2}$ are located between $\beta$6 and $\alpha$8, $\beta$7 and $\beta$8, and $\alpha$9, respectively, as revealed by X-ray analysis. Ves a 1 also has an open conformation and a catalytic site similar to that of vPLA$_{2}$ from *V. basalis*^18^. Studies have shown that Ves a 1 has surface representation of catalytic triad (S170, D198, and H230) and auxiliary site (F241, Y242, N244, and Q249)^3,19^.

The activities of Ves a 1 are also involved in lipid membrane disruption^3,4^ and haemolysis^20^. phospholipase A$_{1}$s (PLA$_{1}$s) family action binds the phospholipid bilayer and hydrolyses the *sn-1* position of the phospholipid, eventually leading to cell lysis and the formation of lysophospholipids and free fatty acids^5,6,20,21^. Furthermore, recently proposed *in silico* modelling of phosphatidylcholines (PC) with venom PLA$_{1}$s, with the short lid domain and $\beta$9 initially interacting with the phospholipid polar heads. The hydrophobic residues in the $\beta$5 immediately attract the substrate to the active site, which is formed by the S137-N165-H229 residues^22^. Interestingly, Ves a 1 was proposed as an auxiliary binding site to biological lipid membranes (Fig. 1), a conserved region for other hymenoptera venom PLA$_{1}$s and mammalian lipases. The Ves a 1 auxiliary site could support Ves a 1 function since the protein was anchored on the phospholipid membrane surface with the help of a $\beta$5 to promote its catalytic site improving the protein-lipid complex^3,17,22^. However, structural and molecular information about Ves a 1 venom remains unknown, and no specific anti-venom for Ves a 1 was found. As a result, our study attempted to identify critical atomistic information to be used for developing anti-venom for Ves a 1 in the state of chemical drugs remains a challenge for the study.

## Methods

### System preparation

The amino acid sequences of Ves a 1 was taken from Uniprot database (UniProtKB code: P0DMB4) and then submitted to AlphaFold 2^23^ for structure prediction. Using the APBS web service^24^, the protonation states of all ionisable amino acids were assigned at pH 7.40. The ff14SB AMBER force field^25^ and the GAFF2 (generalised AMBER force field)^26^. The TIP3P water^27^ box was embedded in each system with a minimum distance of 10 around the protein surface and the solvation box edge, and the box dimensions were set to approximately 79.00 Å $\times$ 68.00 Å $\times$ 83.00 Å. The 29 sodium and 37 chloride ions were then added at random to neutralise at a physiological concentration of 0.15 M. The added hydrogen atoms and solvent molecules were subjected to 3000 steps of steepest descent (SD) energy minimisation with a total of 10,000 steps of minimisation to remove bad atomic contacts, while the rest of the molecules were held fixed. The protein and ligand were then minimised by 3000 SD over a total of 10,000 steps with constrained solvent molecules. Finally, the entire complex was minimised using the same method. The predicted free-ligand Ves a 1 was then simulated using molecular dynamics (MD) for 300 ns by AMBER16^28^. The initial structure of Ves a 1 was then chosen as an open conformations for analysis by free energy landscape (FEL).

### Virtual screening by molecular docking

The available ZINC 15 database^29,30^ and e-Drug 3D database^31^ were filtered converted into the PDBQT format, which is compatible with Autodock Vina version 1.2.0^32^, using OpenBabel^33^. Then, compound structures were performed in databases at the same time for screening with the open conformation Ves a 1, and the script for running the compounds was written by hand in Bash scripting. During the procedure, the protein structure was conFig.d as a rigid molecule with a flexible ligand. To designate the docking location on the protein target, an approximately grid box of 22.00 Å $\times$ 17.00 Å $\times$ 17.00 Å for the catalytic site and 16.00 Å $\times$ 20.00 Å $\times$ 13.00 Å for the auxiliary site was used, with the grid spacing at binding sites of each protein structure. The docking procedure was carried out using 1,2-dimyristoyl-*sn*-glycero-3-phosphocholine (DMPC) (PubChem CID 5459377) as a control, which is a phospholipid commonly found in mammalian cell membrane^18^. The best predicted structure was used as a starting coordinate for MD simulation after competition.

### Molecular dynamics (MD) simulation

Prior to performing MD simulations, the partial atomic charges of the top five drug candidates were calculated using the restrained electrostatic potential (RESP) method at the HF/6-31G* level of theory with the Gaussian09 programmme^34^. Then, using the virtual screening, complexes of Ves a 1 with the control and the top five drug candidates were chosen as the initial conformation for MD simulation. The MD simulations were performed with the PMEMD.cuda module from the AMBER16 package^28,35^. The system was equilibrated in a constant number (N), volume (V), and temperature (T) (NVT) ensemble after it was minimised to remove unusual inter-atomic contacts. The temperature in each simulated system was gradually increased from 0.01 to 310.00 K over 200 ps while a harmonic positional restraint of 10 kcal mol$^{-1}$ Å$^{-2}$ was applied to the C$_{\alpha }$ atoms of protein. The particle mesh Ewald summation method was used to treat electrostatic interactions, with a cut-off distance of 9.00 Å  for non-bonded interactions. All covalent bonds involving hydrogen atoms were constrained using the SHAKE algorithm^36^. Throughout the MD simulation, a simulation time step of 2 fs was used. The Langevin thermostat45 with a collision frequency of 1 ps-1 and the Berendsen barostat^37^ with a pressure-relaxation time of 5 ps were used to control the temperature and pressure. To equilibrate the system, each complex was subjected to four steps of restrained MD simulations at 310.00 K with harmonic restraints of 5.00, 2.50, 1.00, and 0.10 kcal mol$^{-1}$ Å$^{-2}$ for a total of 800 ps, followed by another 200 ps with no restraint. Following that, each simulated system was run under the periodic boundary condition with the isothermal-isobaric of constant number (N), pressure (P), and temperature (T) (NPT) ensemble at 310.00 K and 1 atm pressure, similar to previous studies^38–42^ until it reached 300 ns without restraint. The root-mean-square displacement (RMSD), solvent-accessible surface area (SASA), number of atomic contacts (#Atom contact), and number of ligand to Ves a 1 hydrogen bonds (#H-bond) were calculated from the number of Ves a 1 atoms within 3.50 Å  of each drug. The distance between the hydrogen donor (HD) and acceptor (HA) of less than or equal to 3.50 Å  and the HD-H$\cdots$HA angles of more than or equal to 150.00$^{\circ }$ were used to calculate the H-bond interactions. AMBER16’s CPPTRAJ module and LigandScout software^43^ were used to examine all trajectory analyses and pharmacophore interaction maps, respectively. The total binding free energy ($\Delta$G$_{Bind}^{SIE}$) was calculated using the solvated interaction energy (SIE) method, which was calculated as the following Eq. (1)^44,45^.

1 $$\begin{aligned} \Delta G_{Bind}^{SIE} = \alpha (\underset{Nonpolar term}{\underbrace{\Delta E_{vdW}+\gamma \Delta SA}}+\underset{electrostatic term}{\underbrace{\Delta E_{Ele}+\Delta G_{RF}}})+C \end{aligned}$$

where $\Delta E_{vdW}$ is van der Waals, $\gamma \Delta SA$ is cavity, $\Delta E_{Ele}$ is electrostatic, and $\Delta G_{RF}$ is reaction field. $\Delta E_{vdW}$ and $\Delta E_{Ele}$ are denoted as intermolecular energies in the bound state. The coefficients in every calculation are $\alpha$, $\gamma$, and *C* as 0.105, 0.013, and -2.89, respectively.

### Free energy landscape (FEL) calculation

The free energy landscape (FEL) of Ves a 1 was calculated along with distance and the RMSD of T83-E93 and G251-C261 loops throughout the MD simulation. trial version

2 $$\begin{aligned} \Delta G_{FEL}(x,y)=k_{b}Tln(g(x,y)) \end{aligned}$$

where $k_{b}$ is the Boltzmann constant, *T* is the simulation temperature at 310.00 K, and *g*(*x*, *y*) is the normalised joint probability distribution. The minimum energy was set to zero. A bin size of 0.10 Å was used for the RMSD and distance. A total of 30,000 frames of 300 ns were used for the FEL calculations.

## Results and discussion

### Structure and open/close conformation prediction of Ves a 1

**Figure 2:**
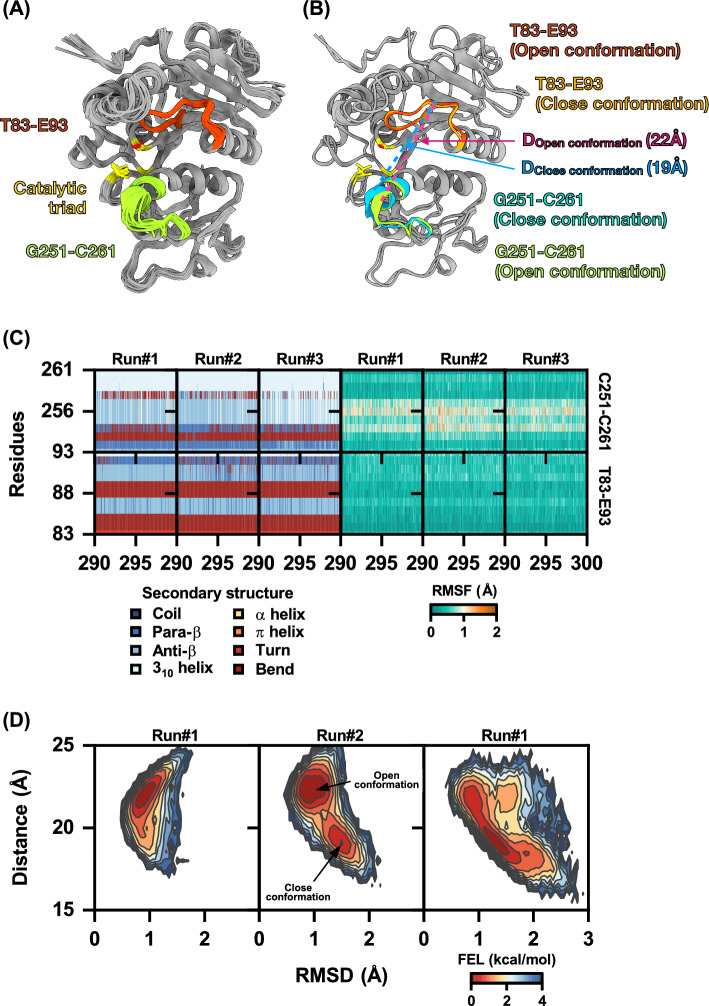
(**A**) The structural superimposition of Ves a 1 from 290 to 300 ns, the G251-C261, T83-E93 loops, and the catalytic triad are shown in orangish-red, greenish-yellow, and yellow, respectively. (**B**) The open and close conformation structures of Ves a 1, with the average distance values between T83-E93 and G251-C261 loops of the two conformations being approximately 22.00 Å  of open conformation and 19.00 Å  of close conformation. (**C**) Time evolution of Ves a 1’s secondary structural elements (left). The heat map of Ves a 1 RMSF profiles (right), with the highest to lowest RMSF shaded from dark orange to viridian, respectively. (**D**) The free energy landscape (FEL) of free-ligand Ves a 1 was calculated for each T83-E93 and G251-C261 loop’s distance vs. RMSD. The distance of catalytic gates (T83-E93 to G251-C261 loops) was measured to be approximately 22.00 Å  and 19.00 Å  for open conformation (D$_{Open~conformation}$) and close conformation (D$_{Close~conformation}$), respectively, and the highest to lowest FEL are gradually shaded from dark blue to dark orange colour. The FEL was calculated by AMBER16’s CPPTRAJ module and plotted using the OriginPro (trial version) software (https://www.originlab.com) as mentioned in FEL calculation method.

The structure prediction was predicted by Alphafold 2^23^. The predicted structure from Alphafold 2 was then uploaded to MolProbity web services^46^ to validate the predicted structures, which produced the Ramachandran plots, which were superimposed to vPLA$_{2}$ of *V. basalis* (PDB code: 4QNN), as shown in Figs. 1 and S2. The Ramachandran plot contained 96% of the preferred region, 100% of the allowed region, and 0% of the disallowed region (Fig. S2), and the RMSD of predicted Ves a1 compared to X-ray structure of vPLA$_{2}$ was 0.93 Å. The time evolution of the secondary structural elements of T83-E93 and G251-C261 loops (Fig. 2C right) showed that T83-E93 and G251-C261 loops were stable during simulation (300 ns, three-replicate), and root mean square fluctuation (RMSF) of T83-E93 and G251-C261 loops (Fig. 2A,C right) showed high average values of fluctuations about 0.89 ± 0.23 Å, 0.96 ± 0.23 Å, and 0.87 ± 0.20 Å. This RMSF result may support the substrate recognition and substrate selectivity functions mentioned previously for G251-C261 loops, and this loop may act as a gate at the catalytic site as transitions between open and close conformations.

To gain a better understanding of the energetic changes associated with the conformational change of Ves a 1, the free energy landscape (FEL) was calculated as a function of distance and RMSD^47,48^ of the T83-E93 and G251-C261 loops. The reaction coordinates of T83-E93 and G251-C261 loops were chosen as the reaction coordinates to monitor the structural change in order to obtain a two-dimensional energy landscape map of FEL. Because the T83-E93 and G251-C261 loops of Ves a 1 are superimposed, they are located at the same $\beta$9 loop and lid-domain of vPLA$_{2}$ (PDB code: 4QNN). The FEL analysis results in Fig. 2D showed that the average distance values between the T83-E93 and G251-C261 loops of the two conformations were approximately 19.00 Å  and 22.00 Å, indicating the close conformation and open conformation, respectively (Fig. 2B). As a result, the time evolution of the secondary structural elements (Fig. 2C left), RMSF, and FEL strongly suggested that the T83-E93 and G251-C261 loops could act as a gate at the catalytic site. The open conformation of Ves a 1 was then chosen as the initial structure for the following steps.

## Results and discussion

### Structure prediction and conformational analysis of Ves a 1

The prediction of the structure of Ves a 1 was performed using Alphafold 2^23^. The predicted structure was then validated using MolProbity web services^46^. The resulting Ramachandran plot revealed that 95.8% of the predicted structure was within the preferred region, 100% was in the allowed region, and 0% was in the disallowed region (see Fig. S2). The predicted Ves a 1 structure was superimposed with vPLA$_{2}$ of *V. basalis* (PDB code: 4QNN), resulting in an RMSD value of 0.93 Å(see Figs. 1, S2).

The stability of the T83-E93 and G251-C261 loops during simulation (300 ns, three-replicate) was confirmed by the time evolution of the secondary structural elements (see Fig. 2C right). The root mean square fluctuation (RMSF) of the T83-E93 and G251-C261 loops also indicated high average values of fluctuations, about 0.89 ± 0.23 Å, 0.96 ± 0.23 Å, and 0.87 ± 0.20 Å(see Fig. 2A,C right). These results support the substrate recognition and substrate selectivity functions of the G251-C261 loops, and suggest that this loop may act as a gate at the catalytic site by transitioning between open and closed conformations.

To further understand the conformational changes of Ves a 1, a free energy landscape (FEL) analysis was performed as a function of distance and RMSD^47,48^ of the T83-E93 and G251-C261 loops. The T83-E93 and G251-C261 loops were chosen as the reaction coordinates to monitor the structural changes, and a two-dimensional energy landscape map of the FEL was obtained. The FEL analysis showed that the average distance values between the T83-E93 and G251-C261 loops of the closed and open conformations were approximately 19.00 Å  and 22.00 Å, respectively (see Fig. 2B,D). This suggests that the T83-E93 and G251-C261 loops could act as a gate at the catalytic site, and supports the notion that the open conformation of Ves a 1 is the initial structure for subsequent steps.

### Potential inhibitors of Ves a 1

The Ves a 1 protein was docked with 2,056 FDA-approved drugs and a ligand control (1,2-Dimyristoyl-*sn*-glycero-3-phosphocholine or DMPC) at both the catalytic and auxiliary sites using AutoDock Vina Version 1.2.0^32^ (Fig. 1A). As the ligand control (DMPC) moved spontaneously from the auxiliary site to the catalytic site during all three individual simulations (Fig. 1B), only the top five drug candidates with catalytic site complexes were selected.

**Figure 3:**
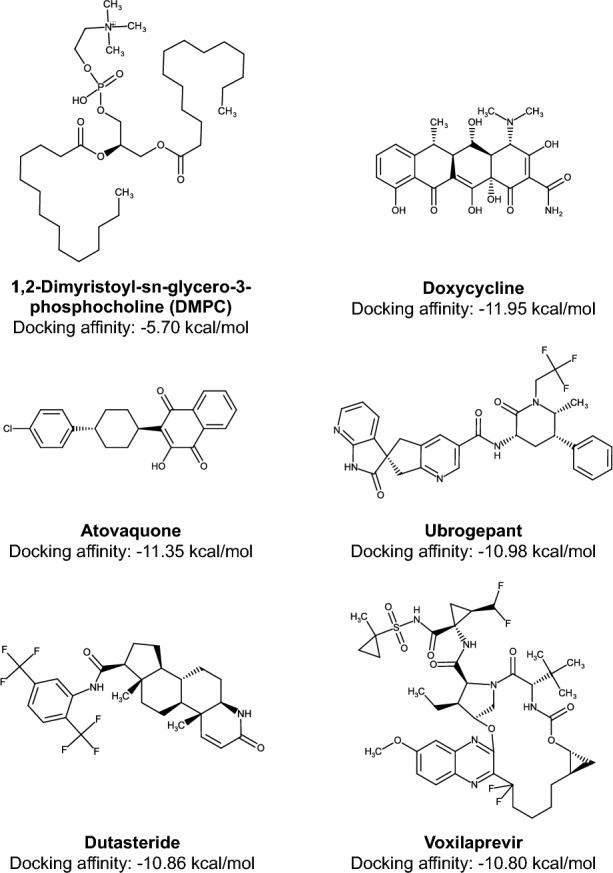
The structures of the top five candidate inhibitors for Ves a 1, along with their respective docking affinity scores as determined by AutoDock Vina Version 1.2.0.

The top five drugs with the highest AutoDock Vina Version 1.2.0 affinity were determined to be $-$ 5.70 kcal/mol of DMPC (PubChem CID 5459377), $-$ 11.95 kcal/mol of doxycycline (ZINC16052277), $-$ 11.35 kcal/mol of atovaquone (ZINC100017856), $-$ 10.98 kcal/mol of Ubrogepant (ZINC95598454), $-$ 10.86 kcal/mol of dutasteride (ZINC3932831), and $-$ 10.80 kcal/mol of voxilaprevir (e-Drug3D ID D1847) (Fig. 3).

### Conformational dynamics of Ves a 1 with DMPC and drugs complex

**Figure 4:**
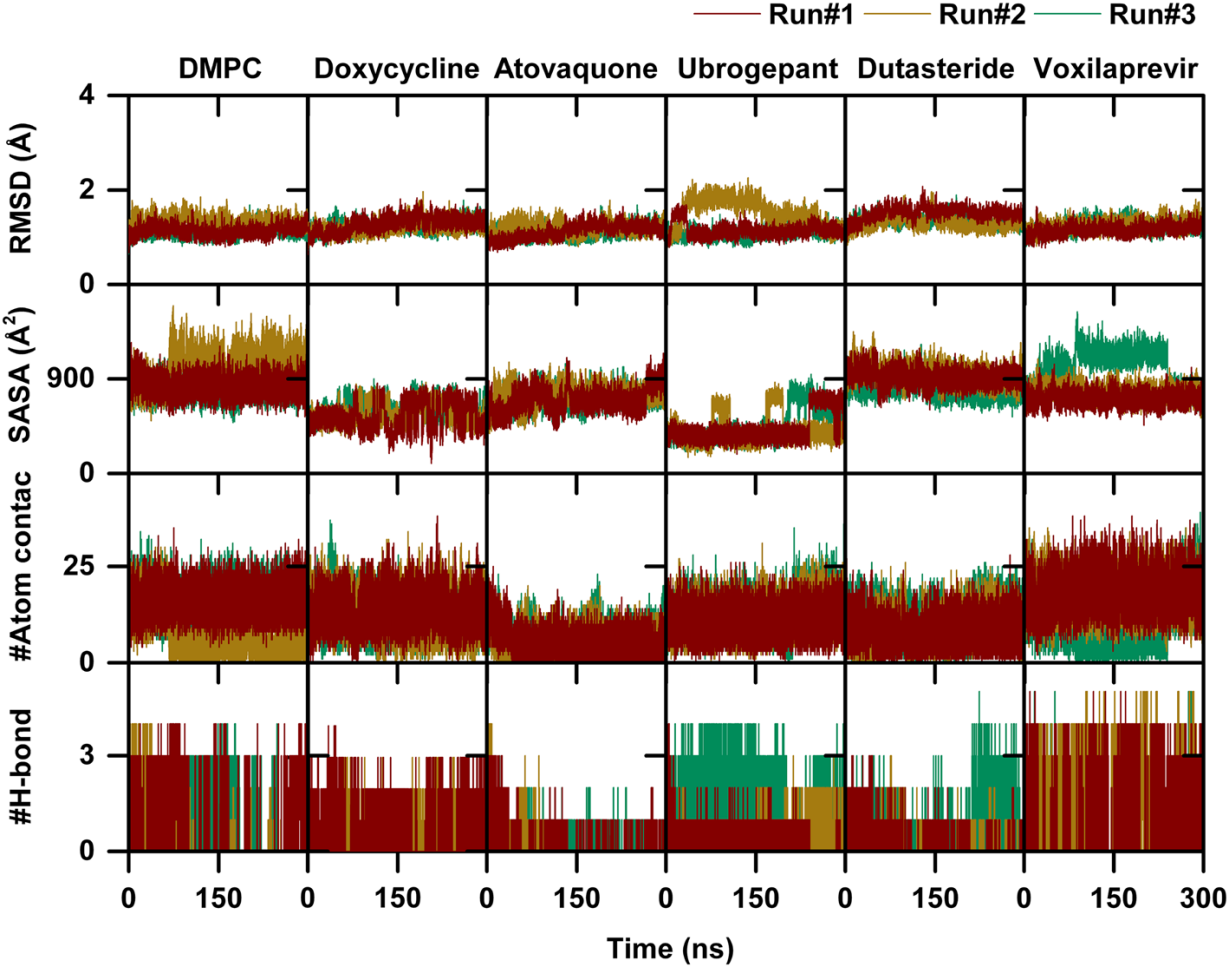
The plots of root-mean-square displacement (RMSD), solvent-accessible surface area (SASA), number of atomic contacts (#Atom contact), and number of hydrogen bonds (#H-bond) of Ves a 1, where the dark red, dark gold, and sea green lines represent the data obtained from the three individual simulations.

The docking affinity scores of the top five Ves a 1 inhibitor candidates, including the control (DMPC), were evaluated using the AutoDock Vina Version 1.2.0. The ligand control and the drug candidates doxycycline, atovaquone, ubrogepant, dutasteride, and voxilaprevir interacted with Ves a 1 at the catalytic site, which was close to the solvent-exposed area. To evaluate the system’s stability, the CPPTRAJ module was used to measure several parameters such as root-mean-square displacement (RMSD), solvent-accessible surface area (SASA), number of atomic contacts (#Atom contact), and number of hydrogen bonds (#H-bond) during the simulation (Fig. 4). All complexes showed high deviation in RMSD values for the first 30 ns and then fluctuated between 1.00 and 1.50 until the end of the simulation. The last 10 ns represented the equilibrium phase in each individual simulation (Run #1, Run #2, and Run #3). This phase was crucial for measuring the analysing of ligand control and the drug candidates. As the catalytic site of Ves a 1 is near the solvent-exposed area, water molecules may play a role in protein-ligand interactions. SASA calculations were performed on the residues within 5.00 Å of each ligand control and drug candidates to characterise the effect of water accessibility at the catalytic site of Ves a 1. The averaged SASA values were measured during the equilibrium phase of each individual simulation (Run#1, Run#2, and Run#3), the order of SASA that referred to the region of catalytic site surface that is exposed sufficiently to interact with solvent molecules was DMPC (905.83 ± 165.61 Å$^2$) > dutasteride (893.02 ± 111.77 Å$^2$) > atovaquone (789.25 ± 86.69 Å$^2$) > voxilaprevir (727.11 ± 57.25 Å$^2$) > doxycycline (557.21 ± 106.52 Å$^2$)> ubrogepant (539.25 ± 139.57). Moreover, the results showed that voxilaprevir had the highest values of #Atom contact and #H-bond, with average values of around 17–19 and around 2, respectively. The order of susceptibility to Ves a 1 among the drug candidates and DMPC was voxilaprevir> DMPC> doxycycline> ubrogepant> dutasteride > atovaquone. These findings suggest that voxilaprevir may be more effective in inhibiting Ves a 1 than the other drug candidates.

### Binding affinity of of Ves a 1 with DMPC and drugs complex

The susceptibility of ligands (doxycycline, atovaquone, ubrogepant, dutasteride, and voxilaprevir) to Ves a 1 was estimated using the solvated interaction energy (SIE) method on 100 snapshots extracted from the last 10 ns of simulation.

Table 1 shows the ranking of the binding free energy $\Delta G_{Bind}^{SIE}$ (Run#1, Run#2, Run#3) from best to worst for voxilaprevir ($-$ 10.16 ± 0.43, $-$ 9.81 ± 0.74, $-$ 10.31 ± 0.40 kcal/mol) > ubrogepant ($-$ 7.48 ± 0.35, $-$ 7.52 ± 0.32, $-$ 7.32 ± 0.35 kcal/mol) > doxycycline ($-$ 6.97 ± 0.46, $-$ 6.34 ± 0.41, $-$ 7.03 ± 0.49 kcal/mol) > dutasteride ($-$ 6.29 ± 0.39, $-$ 6.39 ± 0.62, $-$ 7.07 ± 0.35 kcal/mol) > atovaquone ($-$ 5.89 ± 0.40, $-$ 6.59 ± 0.32, $-$ 5.88 ± 0.58 kcal/mol) and ligand control, DMCP ($-$ 9.49 ± 0.43, $-$ 7.74 ± 0.64, $-$ 9.34 ± 0.51 kcal/mol). The findings suggest that voxilaprevir may have a 1.10-fold (SIE) higher binding affinity to Ves a 1 than DMPC.

**Figure 5:**
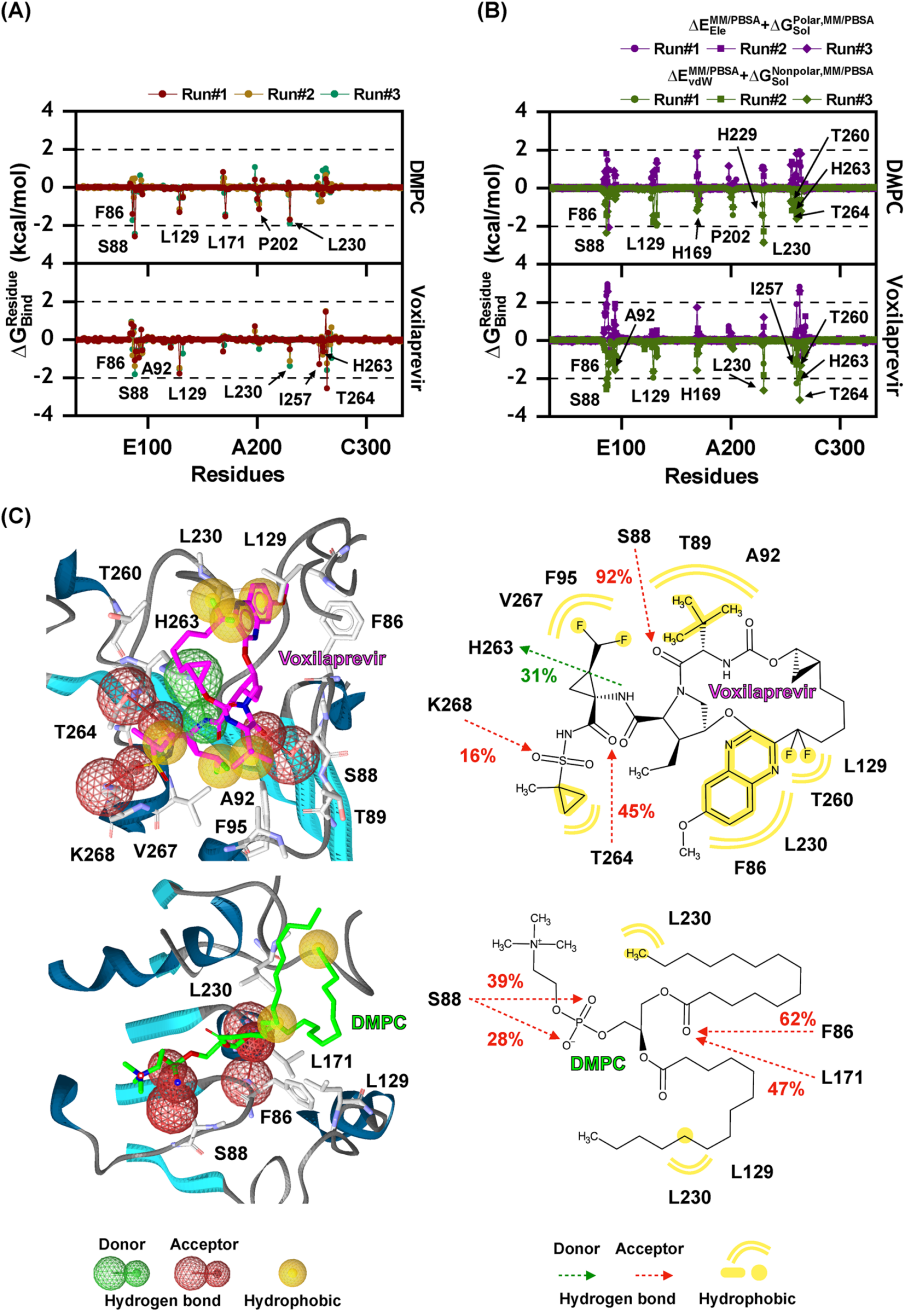
(**A**) The $\Delta$G$_{Bind}^{Residue}$ values of DMPC (top) and voxilaprevir (bottom) in complex with Ves a 1, the contributing residues involved in ligand binding are coloured according to their $\Delta$G$_{Bind}^{Residue}$ values, where the dark red, dark gold, and sea green lines represent the data obtained from the three individual simulations. (**B**) The energy contribution of DMPC (top) and voxilaprevir (bottom), where the vdW ($\Delta E_{vdW}^{MM/PBSA}$+$\Delta G_{Sol}^{Nonpolar,MM/PBSA}$) and electrostatic ($\Delta E_{Ele}^{MM/PBSA}$+$\Delta G_{Sol}^{Polar,MM/PBSA}$) energies are represented in leaf green line and purple line, respectively, the circle, square, and rhombus shapes illustrate the data obtained from the three individual simulations. (**C**) Pharmacophore models and interaction maps with percentage of occurrence in the last 10 ns, calculated by LigandScout software^43^ of of Ves a 1 at catalytic site that contributed to the binding of DMPC (top, left and right) and voxilaprevir (bottom, left and right), where hydrogen bond and hydrophobic interaction are presented as green/red spheres and dashed-arrows, and yellow sphere, respectively.

The MM/PBSA method was used to calculate $\Delta G_{Bind}^{Residue}$ for key residues of voxilaprevir and DMPC, as shown in Fig. 5. Figure 5A,B depict the total energy contribution from each residue associated with ligand binding in both complexes, while Fig. 5C illustrates the drug orientation in the catalytic site of Ves a 1. Only residues with an energy stabilisation of lower than $-$ 1.00 kcal/mol were considered. The results showed that six residues (F86, S88, L129, L171, P202, and L230) and eight residues (F86, S88, A92, L129, L230, I257, H263, and T264) were important for voxilaprevir and DMPC binding, respectively. Voxilaprevir and DMPC have the potential to interact with the catalytic pocket near the catalytic triad, S170, D198, and H263. The main energy contribution for stabilising voxilaprevir and DMPC could be electrostatic contribution in the range of approximately $-$ 1.00 to 3.00 kcal/mol and vdW energy in the range of approximately less than $-$ 2.00 kcal/mol, respectively.

The formation of hydrogen bonds between voxilaprevir and DMPC and their surrounding residues in the catalytic site of Ves a 1 may play a role in the inhibition of this targeted enzyme (Fig. 5C).

**Table 1:** Energy components (kcal/mol) calculated with solvated interaction energy (SIE) method (mean ± SD).

Ligands	$\Delta$E$_{vdW}$	$\Delta$E$_{Ele}$	$\Delta$G$_{RF}$	$\gamma \Delta SA$	$\Delta G_{Bind}^{SIE}$
DMPC					
Run#1	− 56.20 ± 3.76	− 14.40 ± 2.78	19.37 ± 2.36	− 11.77 ± 0.50	− 9.49 ± 0.43
Run#2	− 41.97 ± 6.09	− 3.80 ± 3.67	9.24 ± 2.86	− 9.80 ± 1.44	− 7.74 ± 0.64
Run#3	− 53.36 ± 3.98	− − 19.14 ± 4.78	22.13 ± 3.91	− 11.21 ± 0.70	− 9.34 ± 0.51
Voxilaprevir					
Run#1	− 58.80 ± 3.60	− 84.00 ± 6.63	84.88 ± 4.98	− 11.45 ± 0.32	− 10.16 ± 0.43
Run#2	− 57.96 ± 4.59	− 72.34 ± 11.32	75.11 ± 8.70	− 10.91 ± 0.63	− 9.81 ± 0.74
Run#3	− 60.10 ± 3.03	− 83.61 ± 9.49	84.43 ± 8.21	− 11.52 ± 0.40	− 10.31 ± 0.40
Ubrogepant					
Run#1	− 38.75 ± 3.14	− 6.03 ± 2.62	8.99 ± 1.66	− 7.06 ± 0.36	− 7.48 ± 0.35
Run#2	− 43.35 ± 2.75	− 8.04 ± 1.59	15.78 ± 1.31	− 8.55 ± 0.31	− 7.52 ± 0.32
Run#3	− 43.35 ± 2.90	− 5.05 ± 1.48	14.58 ± 1.43	− 8.47 ± 0.40	− 7.32 ± 0.35
Doxycycline					
Run#1	− 34.81 ± 4.02	− 13.00 ± 2.31	15.12 ± 1.46	− 6.25 ± 0.51	− 6.97 ± 0.46
Run#2	− 32.57 ± 3.57	− 5.24 ± 2.28	10.22 ± 1.81	− 5.30 ± 0.41	− 6.34 ± 0.41
Run#3	− 37.39 ± 3.47	− 13.22 ± 3.59	17.78 ± 2.88	− 6.66 ± 0.62	− 7.03 ± 0.49
Dutasteride					
Run#1	− 31.32 ± 2.32	− 5.86 ± 3.71	11.09 ± 1.81	− 6.33 ± 0.32	− 6.29 ± 0.39
Run#2	− 31.73 ± 4.96	0.04 ± 2.21	4.74 ± 2.42	− 6.45 ± 1.09	− 6.39 ± 0.62
Run#3	− 40.28 ± 3.12	− 0.65 ± 1.18	8.26 ± 1.31	− 7.23 ± 0.32	− 7.07 ± 0.35
Atovaquone					
Run#1	− 28.70 ± 3.43	− 56.61 ± 3.56	61.96 ± 3.46	− 5.24 ± 0.51	− 5.89 ± 0.40
Run#2	− 31.84 ± 2.70	− 50.71 ± 2.54	52.94 ± 2.71	− 5.70 ± 0.36	− 6.59 ± 0.32
Run#3	− 25.14 ± 5.03	− 59.24 ± 7.45	60.73 ± 6.84	− 4.88 ± 0.81	− 5.88 ± 0.58

## Conclusion

After screening 2,056 drugs against Ves a 1, we selected the top five candidates for further investigation. We performed all-atom molecular dynamics simulations on these candidates for 300 ns. Then, we used SIE methods to estimate the binding free energy and understand the key binding interactions between voxilaprevir and Ves a 1. Our study identified the critical amino acid residues F86, S88, A92, L129, L230, H263, T264, and K268, which are involved in voxilaprevir’s binding to the catalytic site of Ves a 1. The electrostatic contribution was found to drive the formation of the molecular complex between Ves a 1 and voxilaprevir. Additionally, hydrogen bond formations from pharmacophore models at S88, H263, T264, and K268 were identified as crucial for efficient binding. Therefore, voxilaprevir has the potential to act as a competitive inhibitor at the catalytic site of Ves a 1, opening up new possibilities for developing more effective anti-venom therapeutic agents for Ves a 1. To confirm the efficacy of voxilaprevir as an anti-venom therapeutic agent for Ves a 1, future experiments such as enzyme inhibitor assays and in vitro/in vivo studies may be necessary.

## Supplementary Information

Supplementary Information.

## Data Availability

The datasets used and/or analysed during the current study are available from the corresponding author on reasonable request.

## References

[1] Gwaltney-Brant, S. M. Zootoxins. In *Reproductive and Developmental Toxicology*, 963–972 (Elsevier, 2017).

[2] Spangfort MD (2000). Structure and biology of stinging insect venom allergens. Int. Arch. Allergy Immunol..

[3] Sukprasert S (2013). Purification and structural characterisation of phospholipase A1 (Vespapase, Ves a 1) from Thai banded tiger wasp (*Vespa affinis*) venom. Toxicon.

[4] Hou M-H (2016). Crystal structure of vespid phospholipase A1 reveals insights into the mechanism for cause of membrane dysfunction. Insect Biochem. Mol. Biol..

[5] Jim SY, Wittkowski KM (2003). Inflammatory role of two venom components of yellow jackets (*Vespula vulgaris*): A mast cell degranulating peptide mastoparan and phospholipase A1. Int. Arch. Allergy Immunol..

[6] Yang H, Xu X, Ma D, Zhang K, Lai R (2008). A phospholipase A1 platelet activator from the wasp venom of *Vespa magnifica* (Smith). Toxicon.

[7] Kularatne, S., Gawarammana, I. & De Silva, P. Severe multi-organ dysfunction following multiple wasp (*Vespa affinis*) stings. *Ceylon Med. J.* **48** (2011). 10.4038/cmj.v48i4.3337 15125411

[8] Kularatne K (2014). Fatal acute pulmonary oedema and acute renal failure following multiple wasp/hornet (*Vespa affinis*) stings in Sri Lanka: Two case reports. J. Med. Case Rep..

[9] Volcheck GW (2002). Hymenoptera (apid and vespid) allergy: update in diagnosis and management. Curr. Allergy Asthma Rep..

[10] Herrera C, Leza M, Martínez-López E (2020). Diversity of compounds in *Vespa* spp. venom and the epidemiology of its sting: A global appraisal. Arch. Toxicol..

[11] Yanagawa Y, Morita K, Sugiura T, Okada Y (2007). Cutaneous hemorrhage or necrosis findings after *Vespa mandarinia* (wasp) stings may predict the occurrence of multiple organ injury: A case report and review of literature. Clin. Toxicol..

[12] Ravikiran, S., Manya, S., Baliga, K. & Bhat, K. G. Acute liver injury, rhabdomyolysis and acute renal failure in a toddler due to multiple stings by *Vespa affinis*. *J. Clin. Diagn. Res.* **13** (2019).

[13] Stief TW (2007). Phospholipase A2 activates hemostasis. Drug Target Insights.

[14] Hiu JJ, Yap MKK (2020). Cytotoxicity of snake venom enzymatic toxins: Phospholipase A2 and L-amino acid oxidase. Biochem. Soc. Trans..

[15] Abe T, Sugita M, Fujikura T, Hiyoshi J, Akasu M (2000). Giant hornet (*Vespa mandarinia*) venomous phospholipases: The purification, characterization and inhibitory properties by biscoclaurine alkaloids. Toxicon.

[16] Rungsa P, Peigneur S, Daduang S, Tytgat J (2018). Purification and biochemical characterization of vest1s, a novel phospholipase a1 isoform isolated from the venom of the greater banded wasp *Vespa tropica*. Toxicon.

[17] Carriére F (1998). Structural basis for the substrate selectivity of pancreatic lipases and some related proteins. Biochimica et Biophysica Acta BBA Rev. Biomembr..

[18] Teajaroen W (2020). A role of newly found auxiliary site in phospholipase A1 from Thai banded tiger wasp (*Vespa affinis*) in its enzymatic enhancement: In silico homology modeling and molecular dynamics insights. Toxins.

[19] Wang J (2020). Insight into the functional diversification of lipases in the endoparasitoid *Pteromalus puparum* (hymenoptera: Pteromalidae) by genome-scale annotation and expression analysis. Insects.

[20] Santos LD (2007). Purification, sequencing and structural characterization of the phospholipase a1 from the venom of the social wasp *Polybia paulista* (hymenoptera, vespidae). Toxicon.

[21] Chou C-C, Hou M-H (2008). Crystallization and preliminary X-ray diffraction analysis of phospholipase A1 isolated from hornet (*Vespa basalis*) venom. Acta Crystallogr. Sect. F: Struct. Biol. Cryst. Commun..

[22] Perez-Riverol A, Lasa AM, dos Santos-Pinto JRA, Palma MS (2019). Insect venom phospholipases A1 and A2: Roles in the envenoming process and allergy. Insect Biochem. Mol. Biol..

[23] Jumper J (2021). Highly accurate protein structure prediction with alphafold. Nature.

[24] Jurrus E (2018). Improvements to the APBS biomolecular solvation software suite. Protein Sci..

[25] Maier JA (2015). ff14SB: Improving the accuracy of protein side chain and backbone parameters from ff99SB. J. Chem. Theory Comput..

[26] Wang J, Wolf RM, Caldwell JW, Kollman PA, Case DA (2004). Development and testing of a general amber force field. J. Comput. Chem..

[27] Jorgensen WL, Chandrasekhar J, Madura JD, Impey RW, Klein ML (1983). Comparison of simple potential functions for simulating liquid water. J. Chem. Phys..

[28] Case, D. A. *et al.**Amber 2016 Reference Manual* 1–923 (University of California, 2016).

[29] Sterling T, Irwin JJ (2015). Zinc 15-ligand discovery for everyone. J. Chem. Inf. Model..

[30] Irwin JJ, Sterling T, Mysinger MM, Bolstad ES, Coleman RG (2012). Zinc: A free tool to discover chemistry for biology. J. Chem. Inf. Model..

[31] Douguet D (2018). Data sets representative of the structures and experimental properties of FDA-approved drugs. ACS Med. Chem. Lett..

[32] Eberhardt J, Santos-Martins D, Tillack AF, Forli S (2021). Autodock vina 1.2.0: New docking methods, expanded force field, and python bindings. J. Chem. Inf. Model..

[33] O’Boyle NM (2011). Open babel: An open chemical toolbox. J. Cheminform..

[34] Frisch, M. J. *et al.**Gaussian 09 Revision e.01* (Gaussian Inc., 2009).

[35] Salomon-Ferrer R, Case DA, Walker RC (2013). An overview of the amber biomolecular simulation package. Wiley Interdiscip. Rev. Comput. Mol. Sci..

[36] Ryckaert J-P, Ciccotti G, Berendsen HJ (1977). Numerical integration of the cartesian equations of motion of a system with constraints: molecular dynamics of n-alkanes. J. Comput. Phys..

[37] Uberuaga BP, Anghel M, Voter AF (2004). Synchronization of trajectories in canonical molecular-dynamics simulations: Observation, explanation, and exploitation. J. Chem. Phys..

[38] Saeloh D (2017). Effects of rhodomyrtone on gram-positive bacterial tubulin homologue FtsZ. PeerJ.

[39] Tanawattanasuntorn T (2020). (–)-Kusunokinin as a potential aldose reductase inhibitor: Equivalency observed via AKR1B1 dynamics simulation. ACS Omega.

[40] Rattanaburee T, Tipmanee V, Tedasen A, Thongpanchang T, Graidist P (2020). Inhibition of CSF1R and AKT by (±)-kusunokinin hinders breast cancer cell proliferation. Biomed. Pharmacother..

[41] Suwanhom P (2021). Synthesis, biological evaluation, and in silico studies of new acetylcholinesterase inhibitors based on quinoxaline scaffold. Molecules.

[42] Jewboonchu J (2022). Atomistic insight and modeled elucidation of conessine towards *Pseudomonas aeruginosa* efflux pump. J. Biomol. Struct. Dyn..

[43] Wolber G, Langer T (2005). Ligandscout: 3-D pharmacophores derived from protein-bound ligands and their use as virtual screening filters. J. Chem. Inf. Model..

[44] Thirunavukkarasu MK, Suriya U, Rungrotmongkol T, Karuppasamy R (2021). In silico screening of available drugs targeting non-small cell lung cancer targets: A drug repurposing approach. Pharmaceutics.

[45] Somboon T (2021). Computational study on peptidomimetic inhibitors against SARS-CoV-2 main protease. J. Mol. Liq..

[46] Chen VB (2010). Molprobity: All-atom structure validation for macromolecular crystallography. Acta Crystallogr. D Biol. Crystallogr..

[47] Pisani P, Caporuscio F, Carlino L, Rastelli G (2016). Molecular dynamics simulations and classical multidimensional scaling unveil new metastable states in the conformational landscape of CDK2. PLoS ONE.

[48] Sun J, Li Z, Yang N (2021). Mechanism of the conformational change of the protein methyltransferase SMYD3: A molecular dynamics simulation study. Int. J. Mol. Sci..

