# Supplementary material for: Computational model for lipid binding regions in phospholipase (Ves a 1) from Vespa venom
Source: Sci Rep. 2023 Jun 30;13:10652. doi: 10.1038/s41598-023-36742-9 (PMC10313747; doi:10.1038/s41598-023-36742-9)
Supplement: Supplementary file 1 — Supplementary Information. [file 41598_2023_36742_MOESM1_ESM.docx]

Supplementary Information

**Computational model for Lipid Binding Regions in Phospholipase (Ves a 1) From Vespa Venom**

Nawanwat C. Pattaranggoon^1^, Sakda Daduang^2^, Thanyada Rungrotmongkol^1,3^,

Withan Teajaroen^4^, Varomyalin Tipmanee^5,∗^, and Supot Hannongbua^6,∗^

^1^ Programme in Bioinformatics and Computational Biology, Graduate school, Chulalongkorn University, Bangkok 10330, Thailand

^2^ Division of Pharmacognosy and Toxicology, Faculty of Pharmaceutical Sciences, Khon Kaen University, Khon Kaen 40002, Thailand

^3^ Center of Excellence in Structural and Computational Biology, Department of Biochemistry, Faculty of Science, Chulalongkorn University, Bangkok 10330, Thailand

^4^ Center for Innovation and Standard for Medical Technology and Physical Therapy, Faculty of Associated Medical Sciences of Khon Kaen University, Khon Kaen 40002, Thailand

^5^ Department of Biomedical Sciences and Biomedical Engineering, Faculty of Medicine, Prince of Songkla University, Hat Yai, Songkhla, 90110, Thailand

^6^ Center of Excellence in Computational Chemistry (CECC), Department of Chemistry, Faculty of Science, Chulalongkorn University, Bangkok 10330, Thailand

^∗^V.T.: tvaromya@medicine.psu.ac.th, S.H.: supot.h@chula.ac.th


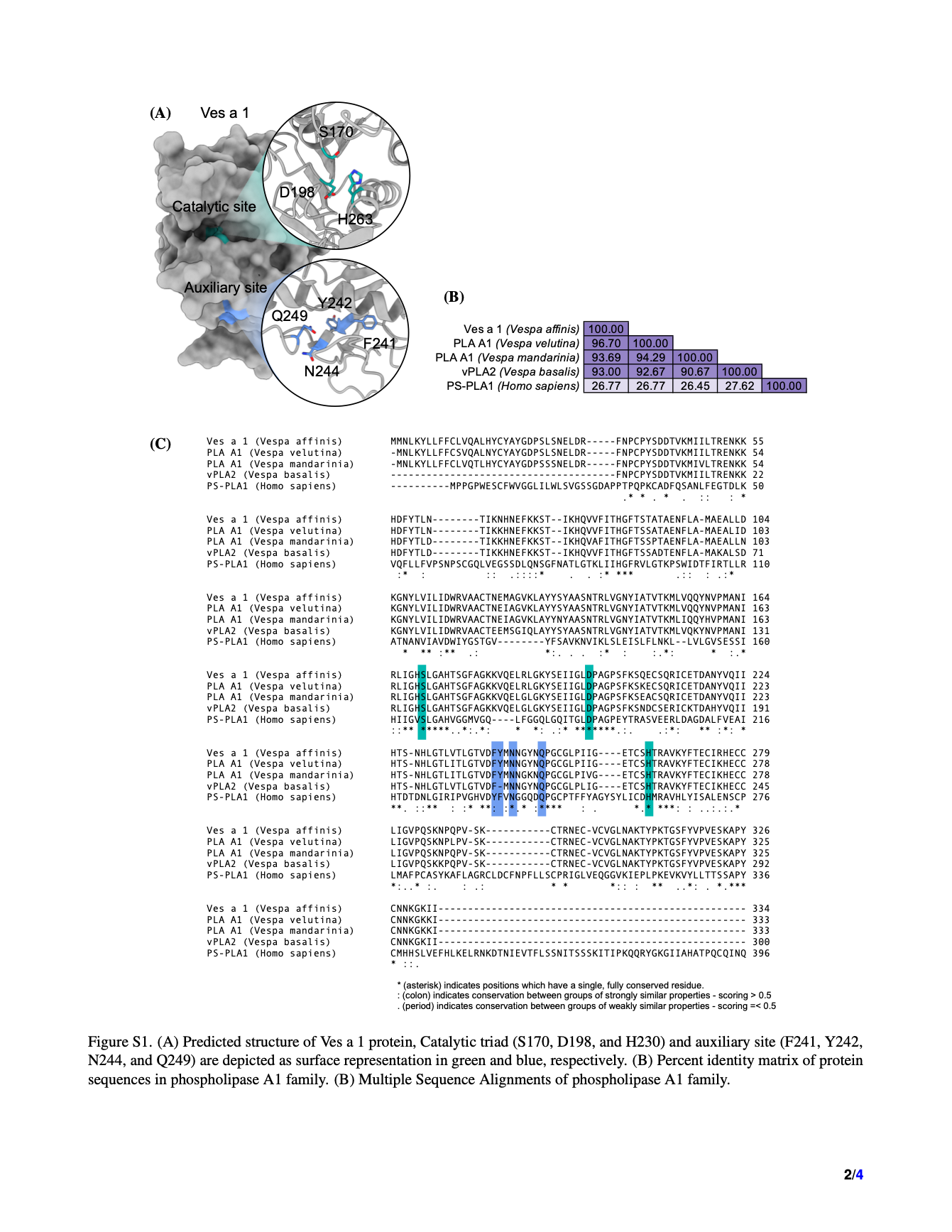


**Figure S1** (A) Predicted structure of Ves a 1 protein, Catalytic triad (S170, D198, and H230) and auxiliary site (F241, Y242, N244, and Q249) are depicted as surface representation in green and blue, respectively. (B) Percent identity matrix of protein sequences in phospholipase A1 family. (B) Multiple Sequence Alignments of phospholipase A1 family.


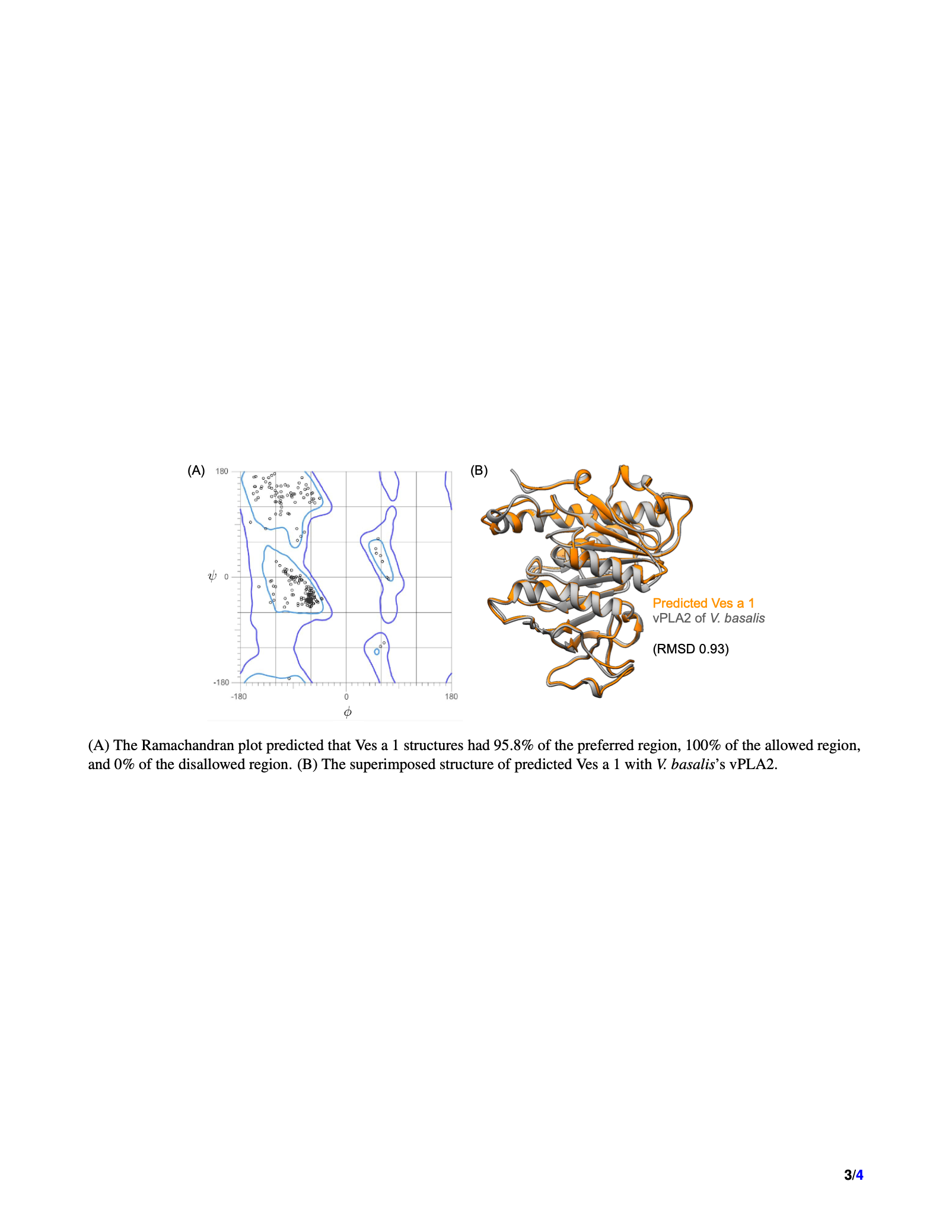


**Figure S2** (A) The Ramachandran plot predicted that Ves a 1 structures had 96% of the preferred region, 100% of the allowed region, and 0% of the disallowed region. (B) The superimposed structure of predicted Ves a 1 with *V. basalis*’s vPLA2.

**Table S1** ∆G_Bind_ values (kcal/mol) of top five drug candidates in complex with Ves a 1 calculated by the MM/PB(GB)SA and SIE Methods (means±standard deviation)

| Energy components | DMPC | | | Atovaquone | | | Dutasteride | | | |
| --- | --- | --- | --- | --- | --- | --- | --- | --- | --- | --- |
|  | Run#1 | Run#2 | Run#3 | Run#1 | Run#2 | Run#3 | Run#1 | Run#2 | Run#3 |  |
| MM/PB(GB)SA-based binding free energy | | | | | | | | | | |
| Gas term | | | | | | | | | | |
| ∆E_vdW_ | -56.35±4.31 | -61.41±6.59 | -51.63±3.84 | -28.97±4.06 | -32.95±2.15 | -24.84±2.62 | -31.61±3.23 | -31.54±4.28 | -40.66±4.50 |  |
| ∆E_ele_ | -30.62±8.88 | -50.67±8.14 | -43.02±12.49 | -127.69±6.83 | -117.10±4.63 | -139.06±17.25 | -15.19±8.39 | 0.81±1.75 | -3.23±2.55 |  |
| ∆E_MM_ | -86.96±7.18 | -112.09±12.05 | -94.65±14.79 | -156.66±7.80 | -150.05±5.22 | -163.90±16.97 | -46.80±7.68 | -30.73±5.07 | -43.88±5.55 |  |
| −TΔS | 31.97±3.82 | 30.11±2.80 | 29.90±3.09 | 19.28±1.60 | 17.85±0.58 | 18.17±2.07 | 23.82±3.35 | 21.15±5.59 | 21.81±1.21 |  |
| Solvation term | | | | | | | | | | |
| ∆G_ele/sol(GBSA)_ | 52.29±8.02 | 73.31±6.40 | 61.85±11.21 | 143.47±5.10 | 129.25±4.69 | 148.76±16.05 | 29.59±5.56 | 12.91±3.49 | 20.46±2.60 |  |
| ΔG_nonpolar/sol(GBSA)_ | -8.64±0.34 | -9.15±0.87 | -7.94±0.49 | -3.93±0.49 | -3.74±0.22 | -3.21±0.47 | -4.71±0.37 | -4.36±0.66 | -4.88±0.34 |  |
| ∆G_sol(GBSA)_ | 43.65±7.95 | 64.16±6.03 | 53.91±10.86 | -156.66±7.80 | -150.05±5.22 | 145.55±15.91 | 24.88±5.49 | 8.55±3.18 | 15.58±2.37 |  |
| ∆G_ele/sol(PBSA)_ | 59.54±6.74 | 81.89±6.57 | 65.45±11.84 | 144.46±6.19 | 131.06±5.03 | 147.20±16.02 | 32.57±5.98 | 12.87±3.79 | 24.14±3.11 |  |
| ΔG_nonpolar/sol(PBSA)_ | -9.24±0.39 | -9.75±0.64 | -8.64±0.33 | -4.49±0.52 | -4.78±0.13 | -4.32±0.40 | -5.13±0.18 | -5.51±0.70 | -6.15±0.26 |  |
| ∆G_sol(PBSA)_ | 50.31±6.77 | 72.14±6.19 | 56.81±11.70 | 139.97±5.98 | 126.28±5.00 | 142.88±15.95 | 27.44±5.88 | 7.35±3.48 | 17.99±2.90 |  |
| Binding free energy | | | | | | | | | | |
| ∆G_bind(MM/GBSA)_ | -11.34±4.71 | -17.81±7.94 | -10.84±5.40 | 2.17±4.60 | -6.69±1.77 | -0.18±3.11 | 1.89±4.34 | -1.03±6.48 | -6.49±4.41 |  |
| ΔG_bind(MM/PBSA)_ | -4.68±5.12 | -9.83±8.29 | -7.94±5.68 | 2.59±3.72 | -5.92±2.92 | -2.86±2.95 | 4.45±4.50 | -2.23±6.69 | -4.08±3.87 |  |
| SIE-based binding free energy | | | | | | | | | | |
| ∆E_vdW_ | -56.20±3.76 | -41.97±6.09 | -53.36±3.98 | -28.70±3.43 | -31.84±2.70 | -25.14±5.03 | -31.32±2.32 | -31.73±4.96 | -40.28±3.12 |  |
| ∆E_ele_ | -14.40±2.78 | -3.80±3.67 | -19.14±4.78 | -56.61±3.56 | -50.71±2.54 | -59.24±7.45 | -5.86±3.71 | 0.04±2.21 | -0.65±1.18 |  |
| ΔG_RF_ | 19.37±2.36 | 9.24±2.86 | 22.13±3.91 | 61.96±3.46 | 52.94±2.71 | 60.73±6.84 | 11.09±1.81 | 4.74±2.42 | 8.26±1.31 |  |
| ΔG_cavity_ | -11.77±0.50 | -9.80±1.44 | -11.21±0.70 | -5.24±0.51 | -5.70±0.36 | -4.88±0.81 | -6.33±0.32 | -6.45±1.09 | -7.23±0.32 |  |
| ΔG_bind_ | -9.49±0.43 | -7.74±0.64 | -9.34±0.51 | -5.89±0.40 | -6.59±0.32 | -5.88±0.58 | -6.29±0.39 | -6.39±0.62 | -7.07±0.35 |  |

**Table S1** (cont.) ∆G_Bind_ values (kcal/mol) of top five drug candidates in complex with Ves a 1 calculated by the MM/PB(GB)SA and SIE Methods (means±standard deviation)

| Energy components | Doxycycline | | | Ubrogepant | | | Voxilaprevir | | |
| --- | --- | --- | --- | --- | --- | --- | --- | --- | --- |
|  | Run#1 | Run#2 | Run#3 | Run#1 | Run#2 | Run#3 | Run#1 | Run#2 | Run#3 |
| MM/PB(GB)SA-based binding free energy | | | | | | | | | |
| Gas term | | | | | | | | | |
| ∆E_vdW_ | -35.77±3.21 | -34.35±2.29 | -34.22±4.53 | -38.26±3.35 | -44.18±2.81 | -42.79±3.46 | -58.36±2.97 | -56.76±2.74 | -58.49±3.72 |
| ∆E_ele_ | -29.92±4.90 | -11.70±3.70 | -26.59±8.11 | -15.88±3.82 | -17.41±2.91 | -10.50±3.89 | -191.66±17.34 | -152.09±22.93 | -192.51±15.63 |
| ∆E_MM_ | -65.69±5.11 | -46.05±4.85 | -60.81±10.80 | -54.14±4.64 | -61.58±4.75 | -53.30±5.69 | -250.02±16.14 | -208.85±24.16 | -251.00±15.69 |
| −TΔS | 26.45±1.59 | 18.52±1.62 | 22.71±2.82 | 26.12±3.16 | 24.80±1.29 | 25.93±2.57 | 28.38±8.89 | 26.86±2.96 | 26.90±5.19 |
| Solvation term | | | | | | | | | |
| ∆G_ele/sol(GBSA)_ | 40.79±4.34 | 26.19±3.16 | 39.86±8.89 | 29.68±2.66 | 37.28±2.82 | 34.13±3.95 | 208.92±14.53 | 173.94±21.94 | 211.84±14.18 |
| ΔG_nonpolar/sol(GBSA)_ | -4.59±0.40 | -3.86±0.17 | -4.16±0.44 | -4.87±0.37 | -5.86±0.28 | -5.77±0.42 | -7.68±0.28 | -7.25±0.33 | -8.00±0.40 |
| ∆G_sol(GBSA)_ | 36.20±4.39 | 22.33±3.13 | 35.70±8.65 | 24.81±2.61 | 31.43±2.68 | 28.36±3.83 | 201.24±14.46 | 166.68±21.71 | 203.83±14.04 |
| ∆G_ele/sol(PBSA)_ | 46.29±4.60 | 31.31±4.12 | 45.01±9.33 | 29.25±3.02 | 45.86±368 | 41.30±5.25 | 218.69±14.87 | 179.75±22.45 | 220.05±14.46 |
| ΔG_nonpolar/sol(PBSA)_ | -5.22±0.26 | -4.52±0.18 | -5.09±0.36 | -5.89±0.33 | -6.55±0.17 | -6.61±0.35 | -8.45±0.18 | -8.18±0.29 | -8.74±0.20 |
| ∆G_sol(PBSA)_ | 41.07±4.56 | 26.79±4.06 | 39.92±9.11 | 23.36±2.93 | 39.30±3.94 | 34.69±5.03 | 210.24±14.91 | 171.57±22.36 | 211.31±14.47 |
| Binding free energy | | | | | | | | | |
| ∆G_bind(MM/GBSA)_ | -3.04±3.92 | -5.20±3.08 | -2.39±5.09 | -3.22±5.03 | -5.35±3.09 | 0.99±3.86 | -20.40±9.57 | -15.30±5.16 | -20.27±6.14 |
| ΔG_bind(MM/PBSA)_ | 1.84±4.00 | -0.74±2.81 | 1.82±5.25 | -4.66±5.17 | 2.52±4.14 | 7.32±4.64 | -11.40±9.90 | -10.42±4.98 | -12.79±5.90 |
| SIE-based binding free energy | | | | | | | | | |
| ∆E_vdW_ | -34.81±4.02 | -32.57±3.57 | -37.39±3.4 | -38.75±3.14 | -43.35±2.75 | -43.35±2.90 | -58.80±3.60 | -57.96±4.59 | -60.10±3.03 |
| ∆E_ele_ | -13.00±2.31 | -5.24±2.28 | -13.22±3.59 | -6.03±2.62 | -8.04±1.59 | -5.05±1.48 | -84.00±6.63 | -72.34±11.32 | -83.61±9.49 |
| ΔG_RF_ | 15.12±1.46 | 10.22±1.81 | 17.78±2.88 | 8.99±1.66 | 15.78±1.31 | 14.58±1.43 | 84.88±4.98 | 75.11±8.70 | 84.43±8.21 |
| ΔG_cavity_ | -6.25±0.51 | -5.30±0.41 | -6.66±0.62 | -7.06±0.36 | -8.55±0.31 | -8.47±0.40 | -11.45±0.32 | -10.91±0.63 | -11.52±0.40 |
| ΔG_bind_ | -6.97±0.46 | -6.34±0.41 | -7.03±0.49 | -7.48±0.35 | -7.52±0.32 | -7.32±0.35 | -10.16±0.43 | -9.81±0.74 | -10.31±0.40 |
